# Proteins Reprogramming: Present and Future

**DOI:** 10.1100/2012/453185

**Published:** 2012-11-22

**Authors:** Yang Yang, Bin Liu, Jianwen Dong, Liangming Zhang, Mao Pang, Limin Rong

**Affiliations:** Department of Spine Surgery, The Third Affiliated Hospital, Sun Yat-sen University, Guangzhou 0086-510630, China

## Abstract

Induced pluripotent stem cells (iPSCs) are of great clinical interest for they are derived from one's own somatic cells and have the potential of committed differentiation without immunological rejection after autografting. However, the use of viral and other modified vectors may still cause tumorigenesis due to chromosome insertion mutation, leading to limited practical use. iPSCs generated by reprogramming proteins overcome the potential safety risk and complicated manipulation procedures, thus they own better application prospective, yet some technical difficulties need to be studied and resolved, for instance, low reprogramming efficiency, unclear transduction, and reprogramming mechanism. In this paper, we summarize the current progress of proteins reprogramming technology for generation of iPSCs and discuss the promising efficiency-improved reprogramming methods by proteins plus other kinds of chemical compounds.

## 1. Introduction

Induced pluripotent stem cells (iPSCs) have been considered as the most significant advance in the field of stem cells research since they were generated in 2006. The Japanese scientists Takahashi and Yamanaka reprogrammed fibroblasts by introducing four factors: *Oct3/4*, *Sox2*, *Klf4*, and *c-Myc* using retroviral vectors, thus activating inner reprogramming system, and finally acquiring iPSCs which resemble embryonic stem cells (ESCs) [1, 2]. Currently, iPSCs are not related with any ethics dispute for they are just derived from one's own somatic cells without destroying embryos. Meanwhile, iPSCs can be induced to committed differentiation *in vitro* like ESCs, and their progenies are able to be autotransplanted for the purpose of treating degenerative and injury diseases in animal experiments with the advantage of owning no immunological rejection. All of these superiorities from iPSCs provide new prospects for regenerative medicine [3]. Also iPSCs own enormous application potentials in the research fields like *in vitro* disease modeling, mechanism research, and drug screening [4, 5]. Since the beginning, the use of integration viral vectors, such as lentiviral and retroviral vectors, which possess comparatively high reprogramming efficiency has been a classic method for the generation of iPSCs [6, 7]. However, these manipulations change the structure of genome by integrating other gene sequences, thus may lead to tumorigenesis [8]. In recent years, a lot of modified methods, for instance, nonintegration adenoviral and sendai viral vector [9, 10], plasmid [11], PiggyBac transposon [12], episomal vector [13], and minicircle [14] have been used in the reprogramming procedures, obtaining better safety. These methods yet cannot get rid of all the possible genome integration completely leaving the consequence that tumorigenesis may still exist. iPSCs induced by reprogramming proteins possess excellent safety and convenience for they are not involved with any change of cellular genome. During the whole proteins reprogramming processes, the main difficulty is how to transport proteins across cytomembrane, thus initiating reprogramming procedures and increasing its low reprogramming efficiency. This paper describes the structure, function, and application of reprogramming proteins transduction vectors; it also discusses other kinds of proteins transduction vectors and chemical compounds that may improve induction efficiency during cellular reprogramming in the future.

## 2. Polyarginine Peptide and Its Application **** During Reprogramming

Cell penetrating peptide (CPP) is a kind of transduction vectors that can combine with chemical compounds or proteins and transduce them across cytomembrane [15]. Polyarginine peptide is composed of six to twelve arginine residues, of which CPP consists of eleven arginine residues (11R) possessing the topmost transduction capacity [16]. At present, in the field of protein transduction therapy, 11R can transduce p53 protein into tumour cells in order to suppress their growth [17–19], which has the same therapeutic effect as viral transduction. Zhou and Kim et al. both used polyarginine peptide to achieve the success of proteins reprogramming [20, 21] (Table 1).

Zhou et al. fused C-terminus of four reprogramming proteins which were generated by *E. coli* with 11R and gained iPSCs induced by proteins for the first time [20]. During the experiment, they found that within six hours reprogramming proteins could access into fibroblasts with the concentration between 0.5 and 8.0 ug/mL and mainly located in the cell nucleus. It was also found that these proteins could maintain stability for more than 48 hours intracellularly. Zhou et al. hereby designed the following proteins reprogramming protocol. They first cultured mouse embryonic fibroblasts (MEFs) which had *OG2/Oct4-GFP* reporter gene with mouse embryonic stem cells medium (mES medium) containing *Oct4*, *Sox2*, *Klf4*, *c-Myc* reprogramming proteins overnight. The concentration of each kind reprogramming protein was 8 ug/mL, and valproic acid (VPA) was added to the culture medium. Then the culture medium was changed into mES medium without any reprogramming protein or VPA for 36 hours. Zhou et al. performed four cycles of proteins induction mentioned above. Finally they transferred these reprogrammed cells on the feeder cells which had been radiated by gamma ray and cultured with mES medium. To their surprise, they did not find iPSCs-like cell colonies until 30 days later. Zhou et al. observed that when using four kinds of reprogramming proteins and VPA, they obtained three GFP (+) cell colonies per 50000 cells; when using three kinds of reprogramming proteins (*Oct4*, *Sox2*, *Klf4*) and VPA, only one GFP (+) cell colony was acquired per 50000 cells; when using reprogramming proteins only, none GFP (+) cell colony was gained. These GFP (−) cell colonies were stained positive for alkaline phosphatase staining (AP staining), demonstrating that these cells were partly reprogrammed at least.

Kim et al. also successfully applied polyarginine peptide and acquired human iPSCs derived from human newborn fibroblasts (HNF) using *Oct4, Sox2, Klf4, and c-Myc* reprogramming proteins for the first time [21], and the difference with former reprogramming protocol was that they used CPP made of nine arginine residues (9R). They first got 9R-proteins composites with *myc* label produced by HEK293 cell line, and they found that these composites could penetrate into cells within eight hours, mainly located in cell nucleus. Kim et al. extended incubation time to 16 hours with reprogramming proteins and culture time to six days when using mES medium only. At last, they noticed that when induction procedures were repeated only three or four times, the acquired cell colonies were negative for AP staining; when repeated six times, more cell colonies emerged and about half of them were positive for AP staining.

## 3. Human Immunodeficiency Virus**** Transactivator Protein and Its Application during Reprogramming

Apart from polyarginine peptide, human immunodeficiency virus transactivator protein (HIV-TAT) can also deliver varieties of proteins across cytomembrane with high efficiency and speed. The structure basis of functional TAT is basic amino acid-rich region consisting of amino acid residues from 47 to 57 with positive charge, and this part of TAT is called protein transduction domain (PTD) [22, 23]. The more PTD transduction vectors fused with proteins are used, the higher its transduction efficiency will be [24]. Proteins transduction using TAT occurs in a concentration-dependent manner, achieving maximum intracellular concentrations in less than five minutes, with nearly equal intracellular concentrations between all cells in the transduced population [25].

 Zhang et al. used reprogramming proteins fused with TAT (TAT-RFs) to induce human foreskin fibroblast (HFF) for the first time and gained iPSCs triumphantly [26]. They pretreated HFF with 0.125 mmol/L VPA for 24 hours, then added five kinds of TAT-RFs (*Oct4*, *Sox2*, *c-Myc*, *Klf4*, *Nanog*) to the culture medium (the concentration of each kind was 50 nmol/L). HFF were reprogrammed by proteins for two hours, soon afterwards the culture medium was replaced by HFF medium without TAT-RFs and HFF were treated by it for 48 hours. From 9th to 18th day during reprogramming, 0.125 mmol/L VPA was added to the culture system. Zhang et al. observed iPSCs-like cell colonies since 13th day. Meanwhile, they found that five kinds of TAT-RFs can induce cell colonies more resembling iPSCs than four kinds of TAT-RFs (*Oct4*, *Sox2*, *c-Myc*, *Klf4*), which was proved by more expressed pluripotent marks.

 During the whole experiment process, Zhang et al. found that the efficiency of reprogramming proteins transduction by TAT and 11R were almost the same: both of them were nearly 100% [26]. However, with respect to initiating reprogramming efficiency, TAT-RFs were much better than 11R-RFs, with 0.01% and 0.001%, respectively [21]. They believed that the possible mechanism might root in the fact that higher positive charge of 11R interfered with reprogramming procedures [16, 27, 28].

## 4. The Analysis of Proteins Induction Methods

Currently, the generation efficiency of iPSCs induced by reprogramming proteins was quite low no matter which kind of transduction vectors was used [21, 26]. In order to increase reprogramming efficiency, one kind of DNA methyltransferase and histone deacetylase inhibitors—VPA was added to the culture system in Zhou and Zhang's protocols. It was believed to enhance reprogramming efficiency by more than 100 times in the case of induction by four kinds of proteins [29]. Zhang et al. proved that VPA was indispensable when TAT-RFs were used to reprogramme [26], yet in the protocol used by Kim et al. VPA was not applied. The reason might be that proteins used by Kim et al. were generated by mammalian cells, while in the methods used by Zhou and Zhang et al., the modified reprogramming proteins were expressed by prokaryotic cells. From these facts, it can be speculated that the same kind of proteins expressed by different cells perhaps have different structures and functions. In Kim's protocol, it is seen that incubation time with reprogramming proteins and subsequent culturing time without proteins were both longer than those from Zhou and Zhang's protocols; reprogramming proteins can access into cells continuously and in quantity, thus own more possibilities to activate reprogramming procedures. From the previously mentioned protocols, we can conclude that using more reprogramming proteins can induce more cell colonies resembling ESCs with more pluripotent marks. This fact also correspond with the fact proved by Liao et al. that six factors protocol owns reprogramming efficiency 10.4 times higher than that of four factors protocol [30].

## 5. The Drawbacks of Proteins Reprogramming Methods

Although proteins reprogramming method has evolved for more than three years, it is still on the early development stage. At present, only three kinds of proteins transduction vectors-11R, 9R, and TAT have been used. Its greatest weakness is low reprogramming efficiency, for example, its induction efficiency is 1000 times lower than that of viral or transposon vectors, yet almost the same as that of plasmids and episomal vectors [31]. Both Zhou and Zhang et al. only used VPA, but no other chemical compounds that could increase reprogramming efficiency. However, in recent years, reprogramming efficiency has been lifted to nearly 10% by some new methods [32]. For instance, the use of SB43142 and PD0325901 can improve the efficiency by 100 times; if thiazovivin was added, it can increase by 200 times [33]. Esteban et al. found that vitamin C enhances the efficiency by alleviating cell senescence, accelerating the alteration of gene expressing, promoting the transition of pre-iPSCs colonies to a fully reprogrammed state and so on [34]. Mali et al. reported that butyrate improved the efficiency by 15–51 times when using four or five reprogramming proteins; if *c-Myc* or *Klf4 *were not used, it could improve by 100–200 times [35]. Although what were mentioned above are not applied to the proteins reprogramming method right now, these still possess the role of certain guidance or reference and may need later research.

Currently, the exact mechanisms of transporting proteins across cytomembrane by CPP and cellular reprogramming are not very clear although some research progresses have emerged [15, 36–38]. Wadia et al. reported that based on a transducible TAT-Cre recombinase reporter assay on live cells, TAT-fusion proteins are rapidly internalized by lipid raft-dependent macropinocytosis after an initial ionic cell-surface interaction [39, 40]. Silhol et al. demonstrated that heparan sulfate (HS) proteoglycans are mainly responsible for full-length TAT internalization in cells for cells deprived of HS proteoglycans could hardly achieve proteins transduction by TAT-PTD [41]. Mikkelsen et al. believed that treatment with DNA methyltransferase inhibitors could improve the overall efficiency of the reprogramming process, thus implying that demethylation was closely related with cellular reprogramming [42]. Up to now there still exist lots of unknown need to be explored, for instance, the influences and effects on the establishment and maintenance of pluripotency by varieties of reprogramming factors; how these factors act on cell genome and initiate reprogramming procedures; the influences on reprogramming efficiency by proteins transduction vectors. The settlement of these problems will help us design better proteins transduction protocols for the sake of increasing reprogramming efficiency in the future.

## 6. The Research Progress of ****Other Proteins Transduction Vectors

Along with the deeper research on the mechanisms of proteins transduction across cytomembrane, some other kinds of proteins transduction vectors, such as fruit flies homologous heterosexual transcription factor ANTP, herpes simplex virus type I transcription factor VP22, own efficient PTD which can transduce varieties of proteins into cells [28]. Li et al. reported that TAT transduction could be dramatically increased 1000-fold when complexed with cationic liposomes (lipo-Tat), compared with TAT alone [43]. Based on the fact that the motif structure of TAT PTD is a powerful amphiphilic helix, Ho et al. strengthened the extent of alpha-helix and optimized the distribution of arginine residues and finally gained proteins vector owning more transduction potential than that of TAT PTD *in vitro* and *in vivo*. The structural domain YARAAARQARA possesses the transduction capacity 33 times higher than that of TAT PTD [44]. Hitsuda et al. reported that three arginine residues (3Rs) associated with pyrenebutyrate are an effective kind of protein transduction vector which owns higher transcriptional activities than that of 11R, although its delivery effectiveness is not as powerful as latter [45]. However, all the previously mentioned transduction vectors have not been applied to proteins reprogramming methods nowadays, intensive and systematic experiments should be carried out to investigate whether they can activate reprogramming procedures more efficiently than existing vectors in the later reprogramming research.

## 7. Conclusion

As one rising member of stem cells, iPSCs have exhibited extremely rapid development and tremendous application potential in less than six years. However, many problems still need to be overcome, such as increasing reprogramming efficiency and safety of iPSCs; seeking out stable methodology of committed differentiation; reducing the complexity of manipulation [46, 47]. Nowadays, the proteins reprogramming method is only restricted to fibroblasts, what we need to do is proving whether other kinds of somatic cells can be reprogrammed by proteins. Proteins reprogramming method has opened prospect for iPSCs generation by defined chemical compounds without using any vectors that can interfere with cellular genome. Recently, the methodology of direct conversion owning more convenience has brought new sights to transdifferentiation which do not need to go through the intermediate state of stem cells, thus generating specific mature cells directly [48–50]; however, it still uses viral vectors, so we could imagine whether it can be used some kinds of proteins to accomplish that goal with the benefit of more usage security. In brief, proteins reprogramming method possesses broad prospect, yet technical difficulties still need to be studied seriously in order to be resolved at last.

## Figures and Tables

**Table 1:** Summary of proteins reprogramming methods.

Cell types	Proteins	Vectors	Culture time with proteins	Culture time without proteins	Use of VPA	References
Mouse embryonic fibroblasts (MEF)	Oct4, Sox2, Klf4, (c-Myc)	11R	Overnight (12 hours)	36 hours	Y	[20]
Human newborn fibroblasts (HNF)	Oct4, Sox2, Klf4, c-Myc	9R	16 hours	6 days	N	[21]
Human foreskin fibroblast (HFF)	Oct4, Sox2, Klf4, c-Myc, (Nanog)	TAT	2 hours	48 hours	Y	[26]
